# Assay for Site-Specific Homologous Recombination Activity in Adherent Cells, Suspension Cells, and Tumor Tissues

**DOI:** 10.21769/BioProtoc.5260

**Published:** 2025-04-05

**Authors:** Yuki Yoshino, Shin Kikuta, Natsuko Chiba

**Affiliations:** 1 Department of Cancer Biology, Institute of Development, Aging and Cancer, Tohoku University, Sendai, Japan; 2 Department of Cancer Biology, Tohoku University Graduate School of Medicine, Sendai, Japan; 3 Laboratory of Cancer Biology, Graduate School of Life Sciences, Tohoku University, Sendai, Japan

**Keywords:** Homologous recombination, PARP inhibitor, BRCA1/2, HBOC, Chemosensitivity, Cas9

## Abstract

Homologous recombination (HR) is a major pathway to repair DNA double-strand breaks. Hereditary breast and ovarian cancer syndrome (HBOC) is caused by germline pathogenic variants of HR-related genes, such as *BRCA1* and *BRCA2 (BRCA1/2).* Cancer cells with HR deficiency are sensitive to poly(ADP-ribose) polymerase (PARP) inhibitors. Therefore, accurate evaluation of HR activity is helpful to diagnose HBOC and predict the effects of PARP inhibitors. The direct-repeat GFP (DR-GFP) assay has been utilized to evaluate cellular HR activity. However, evaluation by the DR-GFP assay tends to be qualitative and requires the establishment of stable cell lines. Therefore, we developed an assay to quantitatively measure HR activity called Assay for Site-Specific HR Activity (ASHRA), which can be performed by transiently transfecting two plasmids. In ASHRA, we use Cas9 endonuclease to create DNA double-strand breaks at specific sites in the genome, enabling the targeting of any endogenous loci. Quantification of HR products by real-time PCR using genomic DNA allows HR activity evaluated at the DNA level. Thus, ASHRA is an easy and quantitative method to evaluate HR activity at any genomic locus in various samples. Here, we present the protocols for adherent cells, suspension cells, and tumor tissues.

Key features

• This assay quantitatively evaluates homologous recombination (HR) activity.

• This assay can measure HR activity in adherent cells, suspension cells, and tumor tissues.

• This real-time PCR-based assay does not require a flow cytometer or next-generation sequencer.

## Graphical overview


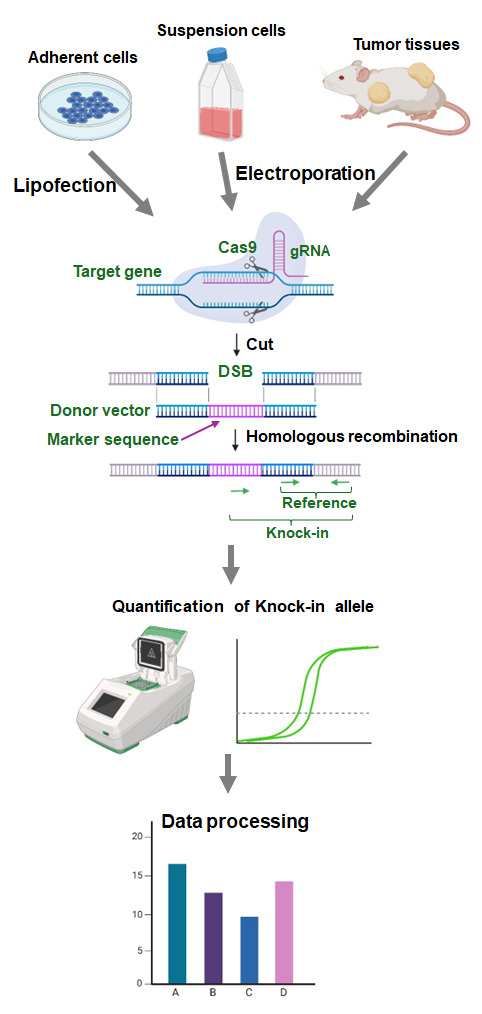


## Background

Homologous recombination (HR) is an essential pathway to repair DNA double-strand breaks (DSBs) and contributes to the repair of interstrand crosslinks. Germline pathogenic variants of HR-related genes, such as *BRCA1* and *BRCA2 (BRCA1/2)*, cause hereditary breast and ovarian cancer syndrome (HBOC) [1]. Sporadic cancers also have alterations of HR-related genes [2]. Cancer cells with HR deficiency are sensitive to cancer treatments, including poly(ADP-ribose) polymerase (PARP) inhibitors, DNA-damaging agents such as platinum agents, and ionizing radiation [3,4]. Thus, accurate evaluation of HR activity is helpful to diagnose HBOC and stratify patients for cancer therapy.

For diagnosis of HBOC and stratification of patients for treatment with PARP inhibitors, genetic tests are performed to detect pathogenic variants of *BRCA1/2* and other HR-related genes in the clinic. However, variants of uncertain significance have been identified, and an accurate functional assay to evaluate HR activity is required for appropriate clinical decision-making [5]. In addition, genomic scarring assays are used to detect HR deficiency in tumor tissues and predict the effects of PARP inhibitors [6] but do not detect changes in HR activity caused by revertant mutations of HR-related genes.

To evaluate cellular HR activity, the direct-repeat GFP (DR-GFP) assay has been utilized [7,8]. However, this assay targets the exogenous GFP locus and detects HR products at the protein level to evaluate HR activity, and its results tend to be qualitative [9]. In addition, the DR-GFP assay requires the establishment of a stable cell line [7,8]. DSB repair can be evaluated by immunostaining for RAD51 (an essential HR factor) or g-H2AX (a DSB marker). However, evaluation of RAD51 foci does not reflect HR activity due to alteration of steps downstream of RAD51 focus formation, and DSBs detected by g-H2AX foci are repaired by non-homologous end-joining in addition to HR [10,11]

**Figure 1.**
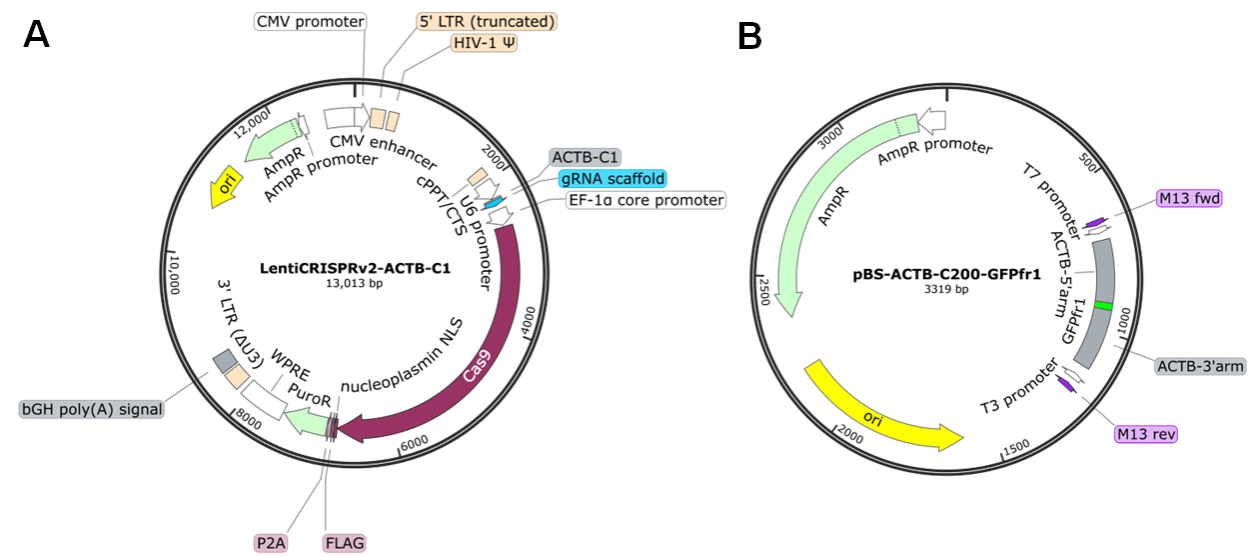
Maps of the plasmids. (A) LentiCRISPRv2-ACTB-C1 plasmid. This plasmid simultaneously expresses gRNA and Cas9 in transfected cells. LentiCRISPRv2-scr has a scrambled sequence instead of the ACTB-C1 gRNA sequence. (B) pBS-ACTB-C200-GFPfr1 plasmid. The priming sequence (GFPfr1) flanked by the donor arms for HR is cloned into the multiple cloning sites of pBlueScript SK II. Plasmid maps were generated with SnapGene software.

To overcome these limitations, we developed an assay to evaluate HR activity called Assay for Site-Specific HR Activity (ASHRA) (Figure 1) [12]. In ASHRA, we use Cas9 endonuclease to create DSBs at specific sites in the genome, enabling the targeting of any endogenous loci. The marker sequence in the donor vector is integrated into the genome during the repair of the DSB by HR. The efficiency of integration of the marker sequence is evaluated by real-time PCR.

Quantification of HR products at the DNA level allows the method to be quantitative [9]. Furthermore, ASHRA can be performed by transiently transfecting two plasmids and does not require the establishment of a stable cell line. We successfully evaluated HR activity in lymphocyte-derived cells and tumor tissues by ASHRA using electroporation for plasmid delivery [13]. The direct measurement of HR activity in tumor tissues accurately predicted sensitivity to PARP inhibitors in vivo [13]. Thus, ASHRA can be performed using a wide range of samples, which greatly widens the application of HR activity evaluation.

Collectively, ASHRA allows easy and quantitative evaluation of HR activity at any locus of interest in the genome. Using ASHRA, we can directly evaluate HR activity in various samples irrespective of the causal genes, type of sequence variations, and shortage of functional information about the variation. In this article, we present the protocols of ASHRA for the measurement of HR activity in adherent cells, suspension cells, and tumor tissues.

## Materials and reagents

**Biological materials**

1. Plasmids for ASHRA [LentiCRISPRv2-ACTB-C1 (Addgene: #169796), LentiCRISPRv2-scr (Addgene: #169795), and pBS-ACTB-C200-GFPfr1 (Addgene: #169798)] (Figure 1) (see General notes for additional information about plasmids)

2. Primers, qPCR grade (Table 1) (see General notes for additional information about primers)

**Table 1.** Primer sets used to detect the knock-in allele (HR product) and reference allele. Sequences are shown in the 5′–3′ direction.

	Forward primer	Reverse primer
**Knock-in allele**	GTCCTGCTGGAGTTCGTGACCG	GTGCAATCAAAGTCCTCGGC
**Reference allele**	AGTTGCGTTACACCCTTTCTTG	

**Reagents**

1. TransIT-X2 dynamic delivery system (Mirus Bio, catalog number: MIR 6000)

2. PEI MAX (Polysciences, catalog number: 24765)

3. Opti-MEM (Thermo Fisher Scientific, catalog number: 11058021)

4. Culture medium and dishes appropriate for the cells used

5. FavorPrep Blood Genomic DNA Extraction Mini kit (Favorgen, catalog number: FABGK 001) or FavorPrep Tissue Genomic DNA Extraction Mini kit (Favorgen, catalog number: FATGK 001)

6. GoTaq qPCR master mix (Promega, catalog number: A6001)

7. NaOH (FUJIFILM Wako, catalog number: 194-18865)

8. Glucose (FUJIFILM Wako, catalog number: 049-31165)

9. Indigo carmine (FUJIFILM Wako, catalog number: 090-00082)

10. FBS (BioWest)

**Solutions**

1. PEI Max solution (see Recipes)

2. Real-time PCR mixture (see Recipes)

**Recipes**

**1. PEI Max solution**

**Table:** 

Reagent	Final concentration	Quantity or Volume
PEI MAX	5 mg/mL	100 mg
1 N NaOH	n/a	Adjust pH to 7.6
H_2_O	n/a	Up to 20 mL
Total	n/a	20 mL

**2. Real-time PCR mixture**

**Table:** 

Reagent	Final concentration	Quantity or Volume
GoTaq qPCR master mix	1×	10 μL
Forward primer (10 μM)	200 nM	0.4 μL
Reverse primer (10 μM)	200 nM	0.4 μL
Template DNA	10–50 ng/reaction	1–5 μL
H_2_O	n/a	Up to 20 μL
Total	n/a	20 μL

**Laboratory supplies**

1. Electroporation cuvettes with 2 mm gap (NEPA Gene, catalog number: EC-002S)

2. 1 mL syringe with a 29G needle (Terumo, catalog number: SS-010F2913T)

3. Hard-shell 96-well PCR plates, low profile, thin wall, skirted (Bio-Rad, catalog number: HSP9601)

4. Microseal B (Bio-Rad, catalog number: MSB1001)

## Equipment

1. CO_2_ incubator for cell culture (any type)

2. Electroporator (NEPA Gene, model: NEPA21 type2)

3. Forceps-type electrode (NEPA Gene, model: CUY650P7)

4. Thermal cycler for real-time PCR (Bio-Rad, model: CFX96)

## Procedure

**A. Preparation of samples**

*Note: See Graphical overview, upper panel.*

ASHRA can measure HR activity in adherent culture cells, suspension culture cells, and tumor tissue masses. The following sections describe the protocols for these different samples.

**A1. Adherent culture cells (e.g., cancer cell lines)**

*Note: See Graphical overview, left of the upper panel.*

1. Seed cells in 3.5 cm dishes.

*Note: The number of cells depends on the cell type. For example, 1 × 10^4^ cells/dish is appropriate for HeLa cells. For slower proliferating cells such as HCC1937 cells, as many as 5–10 × 10^4^ cells/dish may be necessary. The cell number should be adjusted to reach 80%–90% confluency at harvest.*

2. Incubate the cells in a CO_2_ incubator for 24 h.

3. Transfection of siRNA and expression plasmids (if necessary): This step is included in the protocol when knockdown or overexpression of a gene of interest (GOI) is necessary. If such manipulation is not required, proceed to step A1.6.

a. Place 200 μL of Opti-MEM in a microtube for each sample.

b. Add the siRNA and expression plasmid to the microtube.

c. Add TransIT-X2 to the microtube.

d. Mix well and incubate at 37 °C for 30 min.

e. Add the transfection mixture to the cells.

*Notes:*

*1. If you intend to evaluate the effects of knock-down of a GOI on HR activity, it is better to transfect siRNA one day before ASHRA plasmids rather than to perform co-transfection.*

*2. The amount of siRNA and transfection reagent should be titrated for individual experiments. Generally, 10 pmol of siRNA, 0.5 μg of plasmid, and 2–4 μL of TransIT-X2 reagent per 3.5 cm dish are good starting points.*

**Critical:** The transfection reagent used in this step is critical for step A1.6 (transfection of ASHRA plasmids). We usually use the TransIT-X2 dynamic delivery system for siRNA transfection and obtain good results. See Troubleshooting for additional information.

4. Post-siRNA transfection incubation: If you transfected siRNA and/or an expression plasmid, incubate the cells in a CO_2_ incubator for 24 h. If not, proceed to step A1.6.

5. Medium change to fresh growth medium: If you transfected siRNA and/or an expression plasmid, replace the medium with fresh medium prior to transfection of ASHRA plasmids.

6. Transfection of ASHRA plasmids

a. Place 200 μL of Opti-MEM in a microtube for each sample.

b. Add the ASHRA plasmids to the microtube (see Table 2 for plasmid quantities).

**Table 2.** The quantity of plasmid vectors necessary for each type of sample. The Cas9/gRNA plasmid is LentiCRISPRv2-ACTB-C1 or LentiCRISPRv2-scr, and the donor plasmid is pBS-ACTB-C200-GFPfr1. *: dependent on the volume injected into the tumor specimen.

Sample type	Adherent cells	Suspension cells	Tumor tissues
Scale	3.5 cm dish	Cuvette	5 × 5 × 5 mm
Cas9/gRNA plasmid (μg)	0.5	5.0	5–10*
Donor plasmid (μg)	0.5	5.0	5–10*

c. Add 1 μL of PEI Max solution to the microtube.

d. Mix well and incubate at 37 °C for 30 min.

e. Add the transfection mixture to the cells.

*Notes:*

*1. The transfection reagent used in this step is not limited to PEI Max. Any reagent that sufficiently introduces the ASHRA plasmids into the cells can be used.*

*2. LentiCRISPRv2-ACTB-C4 (Addgene: 229849) contains the improved gRNA sequence and can be used as an alternative to LentiCRISPRv2-ACTB-C1. pCG-ACTB-C4 (Addgene: 229846) and pCG-scr (Addgene: 229847) are plasmids in which nonessential sequences are removed from the LentiCRISPRv2 backbone to shorten the plasmids. The pCG series of plasmids can be used as an alternative to LentiCRISPRv2 plasmids.*

7. Post-transfection incubation: Incubate the cells in a CO_2_ incubator for 48–72 h.

*Note: For rapidly proliferating cells such as HeLa cells, 48 h of incubation is sufficient. For slower proliferating cells, longer incubation (e.g., 72 h) may yield better results.*

**A2. Suspension culture cells (e.g., lymphoblastoid cell lines)**

*Note: See Graphical overview, middle of the upper panel.*

1. Collection of cells:

a. Centrifuge cells at 500× *g* at room temperature for 5 min.

b. Remove the supernatant medium.

2. Rinsing of cells with Opti-MEM:

a. Resuspend the cells in 5 mL of Opti-MEM.

b. Centrifuge at 500× *g* for 5 min at room temperature.

c. Remove the supernatant medium.

3. Resuspension and counting of cells:

a. Resuspend the cells in 1–2 mL of Opti-MEM.

b. Count the cell number.

4. Preparation of cells for electroporation:

a. Place 10 × 10^4^ cells in a microtube for each sample.

b. Centrifuge at 500× *g* for 5 min at room temperature.

c. Remove the supernatant medium.

d. Resuspend the cells in 100 μL of Opti-MEM.

5. Electroporation:

a. Add 10 μg of ASHRA plasmids to the microtube. Add the expression plasmid or siRNA for the GOI in this step if necessary.

b. Apply the electroporation pulse.

c. Cool the cells on ice for 1 min immediately after pulse application.

d. Transfer the cells to prewarmed growth medium in a 3.5 cm dish.

*Note: The pulse conditions should be titrated for individual experiments. For human lymphoblastoid cell lines, we use the following conditions with a NEPA21 electroporator and 2 mm gap cuvettes: voltage, 125 V; pulse duration, 2 ms; pulse repeats, six times; polar switch, -.*

6. Post-transfection incubation: incubate the cells in a CO_2_ incubator for 48–72 h.

**A3. Tumor tissue mass**

*Note: See Graphical overview, right of the upper panel.*

1. Transplantation and tumor development:

a. Transplant appropriate cancer cells (e.g., HeLa-tet-shBRCA1 cells [13]) into nude mice or other immunocompromised mice.

b. Allow mice to develop tumors measuring up to 1 cm in diameter.

2. Drug administration: Administer a drug (e.g., doxycycline) if needed. The administration protocol should be determined in each experiment.

3. Tumor specimen preparation:

a. Surgically excise the tumor.

b. Trim the tumor to obtain tumor mass samples (approximately 150 mm^3^) with an approximately cubic shape measuring 5 mm.

4. Plasmid injection and electroporation:

a. Inject 0.1–0.2 mL of the ASHRA plasmid solution (100 μg/mL) into the tumor specimen using a 1 mL syringe with a 29G needle. Multiple injections may be necessary to ensure an even distribution.

b. Rinse the tumor specimen with 5% glucose solution.

c. Apply the electroporation pulse.

*Notes:*

*1. The addition of indigo carmine to the ASHRA plasmid solution at a concentration of up to 1 mg/mL allows the visualization of the injection solution to help ensure it is evenly injected into the specimen [13].*

*2. The pulse conditions should be titrated for individual experiments. For HeLa cell tumors, we use the following conditions with a NEPA21 electroporator and a forceps-type electrode: voltage, 125 V; pulse duration, 5 ms; pulse repeats, two times; polar switch, +*.

5. Incubation: incubate the tumor specimens in DMEM containing 8% FBS or appropriate growth medium in a CO_2_ incubator at 37 °C for 72 h.

*Note: The samples should be immersed in medium immediately after electroporation to prevent cell damage.*

**B. Quantification of knock-in products**

*Note: See Graphical overview, middle panel.*

1. Extraction of genomic DNA: extract genomic DNA from the samples using standard methods.

*Note: The purity of the obtained DNA sample is critical for the next quantification step. It is recommended to use commercial kits that yield PCR-compatible DNA. We usually use the FavorPrep Blood Genomic DNA Extraction kit for culture cells and the FavorPrep Tissue Genomic DNA Extraction kit for tumor tissue masses. However, kits from other manufacturers (e.g., QIAamp DNA kit from Qiagen) also work well. For culture cells, purification using SPRI beads can also be performed [14].*

2. Measurement of DNA concentration: measure the DNA concentration by standard methods (e.g., ultraviolet absorbance or fluorescent dye assay). If there are samples with significantly different DNA concentrations, adjust the concentrations to ensure uniformity.

3. Setup and performance of quantitative PCR (qPCR)

a. Set up qPCR.

b. Perform real-time qPCR using the following PCR program: denaturation at 98 °C for 2 min, followed by 50 cycles of 98 °C for 15 s and 60 °C for 2 min.

c. Gather the Ct values (sample data are shown in Table 3).

*Note: For each sample, both the knock-in primer set and reference primer set should be used. See Recipes for the reaction mixture recipe. Each quantification should include duplicate or triplicate reactions.*

**Table 3.** Ct values from three independent experiments (Figure 1). Ref, reference. KI, knock-in.

siRNA	gRNA	Ct of sample 1		Ct of sample 2		Ct of sample 3	
		**Ref**	**KI**	**Ref**	**KI**	**Ref**	**KI**
control	scr	22.7	35.8	19.4	33.9	19.4	33.2
control	ACTB	23.1	33.8	19.6	29.6	18.9	29.3
RAD51	scr	23.0	36.9	19.0	33.8	19.0	33.7
RAD51	ACTB	22.9	35.6	19.1	31.2	19.2	31.3

**C. Processing of data**

*Note: See Graphical overview, lower panel.*

**C1. Comparisons of samples with homogeneous properties (such as experiments using the same cell line)**

In this situation, transfection efficiency and background amplification of the primer sets can be considered to be similar. Thus, the comparison of HR activity with that in control samples is appropriate to assess HR activities in the samples.

**Figure 2.**
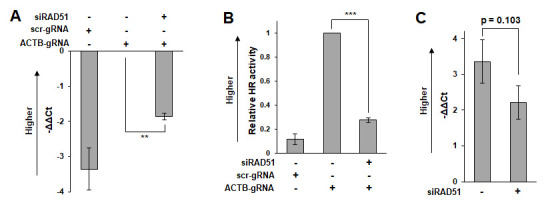
Measurement example. HeLa cells were transfected with siRNA targeting RAD51 (hs.Ri.RAD51.13.1, Integrated DNA Technologies, Coralville, IA, USA) or control siRNA (5′-UUCUCCGAACGUGUCACGUdTdT-3′). One day after transfection of siRNA, the ASHRA plasmids were transfected. After 48 h of incubation following plasmid transfection, genomic DNA was extracted for quantification by real-time PCR. (A) -ΔΔCt values calculated by Formula 1. (B) Relative homologous recombination (HR) activity calculated by Formula 2. (C) -ΔΔCt values calculated by Formula 3. The bars and error bars show the mean and standard error of the mean, respectively. The *p*-values were calculated using a student’s *t*-test.

Calculate the ΔΔCt value according to Formula 1 (Figure 2A). If necessary, convert the ΔΔCt value to the relative knock-in efficiency using Formula 2 (Figure 2B). The Ct value is inversely proportional to the logarithm of the target concentration in the sample. Therefore, the result of Formula 1 represents the logarithm of the ratio of the frequency of the knock-in of marker sequence in sample X to that in the control sample. Thus, this can be converted to a constant by Formula 2.

**Formula 1:** ΔΔCt[sample X] = (Ct[knock-in, sample X] – Ct[knock-in, control sample]) – (Ct[reference, sample X] – Ct[reference, control sample])

**Formula 2:** Relative knock-in efficiency = 2^(-ΔΔCt)

**C2. Comparisons of samples with significantly different properties (such as comparisons of different cell lines)**

In this situation, transfection efficiency and the background signals of samples can vary significantly. Therefore, appropriate normalization of the variation between samples is necessary. To do this, control samples transfected with ACTB/Cas9 and scr/Cas9 should be prepared to subtract the background signals for each sample.

Calculate the ΔΔCt value according to Formula 3 (Figure 2C). Use -ΔΔCt as the representative value of HR activity. The values calculated by Formula 3 tend to be more variable than those calculated by Formulas 1 and 2 (Figure 2C). More repeat experiments may be necessary to obtain statistical significance.

**Formula 3:** ΔΔCt[sample X] = (Ct[knock-in, ACTB, sample X] – Ct[knock-in, scr, sample X]) – (Ct[reference, ACTB, sample X] – Ct[reference, scr, sample X])

## Validation of protocol

This protocol has been used and validated in the following research articles:

• Yoshino et al. [12]. Evaluation of site-specific homologous recombination activity of BRCA1 by direct quantitation of gene editing efficiency. *Scientific Reports* (Figures 4 and 6).

• Endo et al. [9]. BRCA1/ATF1-Mediated Transactivation is Involved in Resistance to PARP Inhibitors and Cisplatin. *Cancer Research Communications* (Figures 1, 2, 4, and 6).

• Motonari et al. [13]. Evaluating homologous recombination activity in tissues to predict the risk of hereditary breast and ovarian cancer and olaparib sensitivity. *Scientific Reports* (Figures 2 and 5).

• Yanaihara et al. [15]. Paclitaxel sensitizes homologous recombination-proficient ovarian cancer cells to PARP inhibitor via the CDK1/BRCA1 pathway. *Gynecologic Oncology* (Figures 1D, 2D, 3C, 3D).

• Iida et al. [16]. Bevacizumab increases the sensitivity of olaparib to homologous recombination-proficient ovarian cancer by suppressing CRY1 via PI3K/AKT pathway. *Frontiers in Oncology* (Figure 2A, E, and F).

## General notes and troubleshooting

**General notes**

1. Cas9/gRNA expression plasmid: In this protocol, the LentiCRISPRv2 plasmid was used as the backbone to express Cas9 and gRNA. However, any plasmid that co-expresses Cas9 and gRNA can be used. It is possible to express Cas9 and gRNA from separate plasmids, although we have not tried this.

2. The donor plasmid was constructed from pBlueScript SK II as the backbone. It is preferable to use plasmids that lack any functions such as transcriptional activity that might affect the HR pathway. Arm length significantly affects the efficiency of knock-in of the marker sequence. The longer the arms are, the more efficient knock-in is. However, arms that are too long make qPCR difficult (see General note 3).

3. Primer design: Primer sets to detect the reference allele are designed to bind near the target locus. One primer to detect the knock-in allele is designed to bind within the marker sequence, while the other primer is designed to bind outside the 5' or 3' arm. Therefore, long arm sequences result in larger amplicon sizes to detect the knock-in allele, which may reduce the amplification efficiency of qPCR.

**Troubleshooting**

1. Low transfection efficiency: If PEI does not provide sufficient transfection efficiency, more efficient transfection reagents may improve transfection efficiency. In our experience, a transfection efficiency of approximately 10% is sufficient to detect HR products. After transfecting siRNA or an expression plasmid for the GOI (step A1.3), the transfection efficiency of ASHRA plasmids decreases. When TransIT-X2 reagent is used as the transfection reagent in step A1.3, a decrease in transfection efficiency of ASHRA plasmids is acceptable. Other reagents such as Lipofectamine RNAiMAX and Lipofectamine LTX significantly decrease the transfection efficiency of ASHRA plasmids, making the assay difficult.

2. Problems with electroporation: If the transfection efficiency and/or cell survival rate are too low, then electroporation conditions such as voltage, pulse duration, number of pulse repeats, and polar switch should be adjusted.

3. Weak/no signal in qPCR: The amount of template genomic DNA required for the reaction varies depending on cell properties. When using HeLa cells, which have sufficient HR activity and high transfection efficiency, it is possible to detect the knock-in allele using as few as approximately 1,000 genome copies (approximately 3 ng) per reaction. However, more genomic DNA may be required when using cells with low HR activity or a low transfection efficiency.

4. Nonspecific amplification in qPCR: The purity of the template genomic DNA is critical. If nonspecific amplification is too high, then repurification of genomic DNA using a kit or ethanol precipitation may improve the reaction. Furthermore, contamination of genomic DNA by the donor plasmid may increase nonspecific amplification. When harvesting cells, carefully remove the medium from the cells to avoid contamination of the donor plasmid. If these measures do not improve qPCR, it may be necessary to redesign the primers.
